# Transmission of NDM-5-Producing and OXA-48-Producing Escherichia coli Sequence Type 648 by International Visitors without Previous Medical Exposure

**DOI:** 10.1128/spectrum.01827-21

**Published:** 2021-12-22

**Authors:** Sohei Harada, Masahiro Suzuki, Toshiharu Sasaki, Aki Sakurai, Masato Inaba, Hosoda Takuya, Mitsutaka Wakuda, Yohei Doi

**Affiliations:** a Department of Infection Control and Prevention, The University of Tokyo Hospital, Hongo, Bunkyo-ku, Tokyo, Japan; b Department of Infectious Diseases, Fujita Health University School of Medicine, Toyoake, Aichi, Japan; c Department of Microbiology, Fujita Health University School of Medicine, Toyoake, Aichi, Japan; d Department of Joint Research Laboratory of Clinical Medicine, Fujita Health University Okazaki Medical Center, Okazaki, Aichi, Japan; e Department of Joint Research Laboratory of Clinical Medicine, Fujita Health University Hospital, Toyoake, Aichi, Japan; f Division of Infectious Diseases, University of Pittsburgh School of Medicine, Pittsburgh, Pennsylvania, USA; National University Hospital

**Keywords:** *Escherichia coli*, NDM-5, OXA-48, ST648, carbapenemase

## Abstract

Carbapenemase-producing Escherichia coli sequence type (ST) 648 strains were isolated from two international visitors without previous medical exposure from Southeast Asian countries in a hospital in Japan. One isolate, FUJ80154, carried *bla*_NDM-5_ in a complex class 1 integron on an IncFIB/FII plasmid; the other isolate, FUJ80155, carried two copies of *bla*_OXA-48_ on the chromosome flanked by IS*1R* on both sides. The core-genome based-phylogenetic analysis with publicly available genome data of E. coli ST648 carrying *bla*_NDM-5_ or *bla*_OXA-48-like_ demonstrated high genetic similarity between FUJ80154 and NDM-5-prooducing E. coli ST648 strains isolated in South and Southeast Asian countries. On the other hand, no closely related isolates of FUJ80155 were identified. In the absence of prior hospitalization overseas, neither patient had qualified for routine screening of multidrug-resistant organisms, and the isolates were incidentally identified in cultures ordered at the discretion of the treating physician.

**IMPORTANCE** Although patients with history of international hospitalization are often subject to screening for multidrug-resistant organisms, it is unclear whether patients who reside in countries where carbapenemase-producing *Enterobacterales* (CPE) is endemic but have no history of local hospitalization contribute to the transmission of CPE. In this study, NDM-5-producing and OXA-48-producing Escherichia coli sequence type (ST) 648, a recently recognized high-risk, multidrug-resistant clone, were detected from two overseas visitors without previous medical exposure. The findings of this study suggest that active surveillance culture on admission to hospital may be considered for travelers from countries with endemicity of carbapenem-resistant organisms even without history of local hospitalization and underscore the need to monitor cross-border transmission of high-risk clones, such as carbapenemase-producing E. coli ST648.

## OBSERVATION

The spread of carbapenemase-producing *Enterobacterales* (CPE) is recognized as a global public health threat since the 2000s (1). CPE may be introduced into a country with low endemicity by patients with a history of hospitalization in a country where they are endemic. It has been reported that the frequency of isolation of CPE in a country may increase sharply when high-risk CPE clones are introduced from abroad and spread across health care facilities in a short period of time (2, 3). Japan is characterized by low rates of CPE, and most of these isolates produce imipenemase (IMP)-type carbapenemases (4, 5). On the other hand, CPE producing Klebsiella pneumoniae carbapenemase (KPC), NDM, or OXA-48-like enzymes are extremely rare in Japan even in recent years, and most are identified in patients with a history of overseas hospitalization (6, 7). Therefore, many major hospitals in Japan screen patients with history of overseas hospitalization for multidrug-resistant organisms (MDROs), including CPE (7).

In this study, we investigated the epidemiological characteristics of patients from whom CPE producing non-IMP enzymes (non-IMP CPEs) was isolated in 2019 in a 1,435-bed Japanese university hospital. This study was approved by the Ethics Committee of Fujita Health University School of Medicine (approval number: HM19-170). Bacterial identification and antimicrobial susceptibility testing in the hospital were performed using Vitek MS and Vitek 2 (bioMérieux, Marcy l'Etoile, France), respectively. Modified carbapenem inhibition method (mCIM) was performed on carbapenem-non-susceptible *Enterobacterales* according to the CLSI M100 guidelines, for the confirmation of carbapenemase production (8). Types of carbapenemases produced by CPE isolates were analyzed with NG-Test CARBA 5 (NG Biotech, Guipry, France).

Only two non-IMP CPE isolates were detected during the study period (Table S1 in the supplemental material). One isolate (FUJ80154) was identified as NDM-producing E. coli and the other isolate (FUJ80155) was identified as OXA-48-like-producing E. coli. Both patients were long-term residents of Southeast Asian countries but had no underlying medical conditions and no history of local hospitalization. The patients were not among the prespecified population for active surveillance culture at the hospital, but cultures were submitted at the discretion of the treating physicians. While FUJ80154 was resistant to all β-lactams, FUJ80155 was susceptible to carbapenems reflecting the relatively lower hydrolytic activity of OXA-48-like enzymes against carbapenems compared with that of NDM enzymes.

Whole genome sequencing of the non-IMP CPE isolates was performed with Illumina MiSeq (Illumina, Inc., San Diego, CA) and MinION (Oxford Nanopore Technologies, Oxford, UK). Hybrid *de novo* assembly using both MiSeq and MinION reads was conducted with Unicycler (version 0.4.9b). Genetic characterization of the genomes was performed using software available at the website of Center for Genomic Epidemiology (https://cge.cbs.dtu.dk/services/) and ClermonTyping (9). Both isolates were E. coli of phylogroup F and sequence type (ST) 648, and carried multiple virulence genes, including those associated with uropathogenicity (*chuA*, *fyuA*, *yfcV*) and iron uptake (*chuA*, *fyuA*, *irp2*, *iutA*, *sitA*) (Table S1 in the supplemental material) (10, 11).

**FIG 1:**
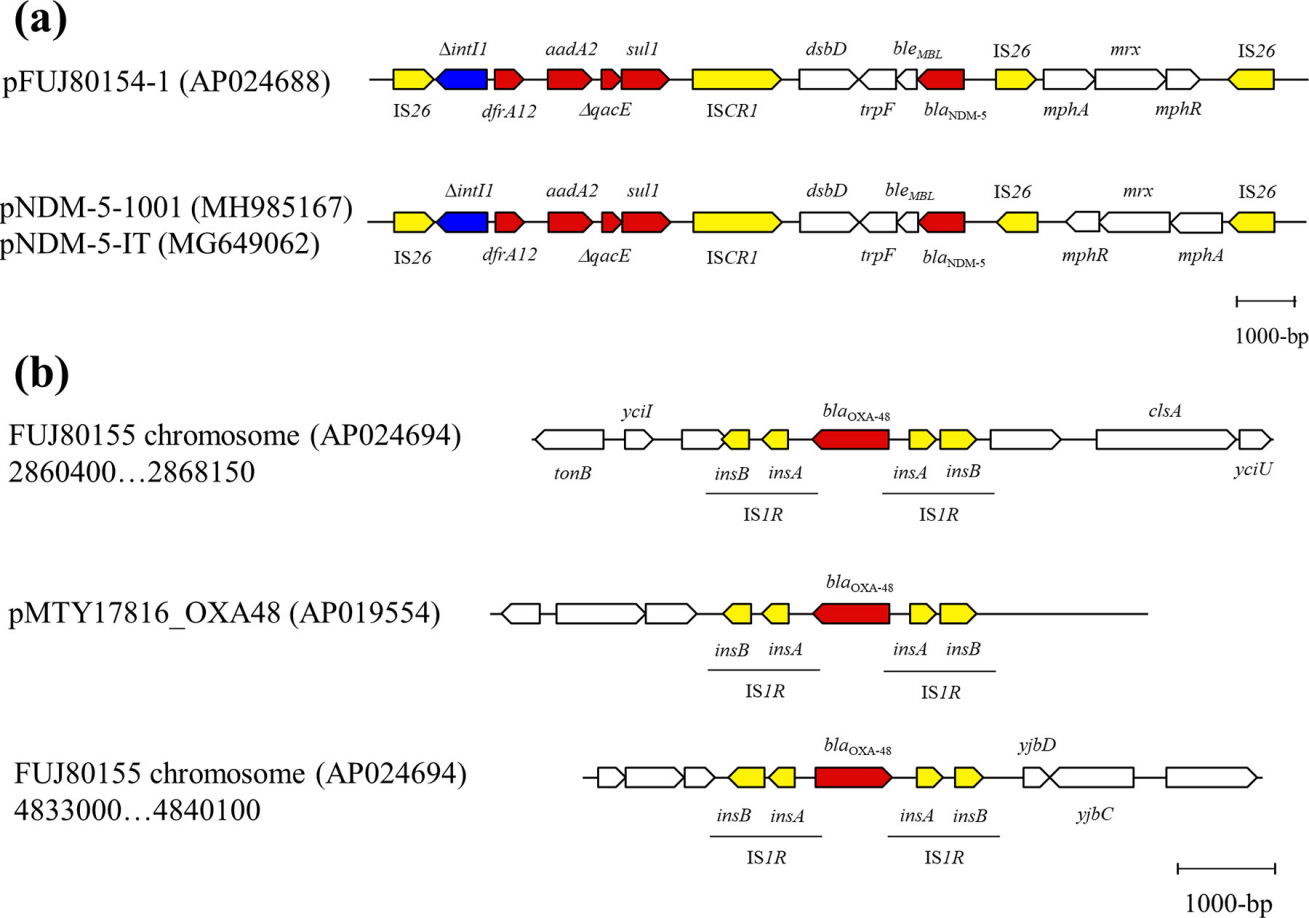
Genetic environment of carbapenemase genes. (a) Genetic environment of *bla*_NDM-5_ on pFUJ80154-1 plasmid and the similar genetic structure identified in previous studies and (b) genetic environment of two copies of *bla*_OXA-48_ on the chromosome of FUJ80155 and the identical genetic structure identified in a previous study. Block arrows indicate confirmed or putative open reading frames (ORFs) and their orientations. Arrow size is proportional to the predicted ORF length. The color codes are as follows: yellow, transposase genes; blue, integrase genes; red, antibiotic resistance genes; white, other.

FUJ80154 possessed *bla*_NDM-5_ on an IncFIB/FII plasmid (pFUJ80154-1) and FUJ80155 carried *bla*_OXA-48_ on the chromosome. Additionally, both isolates possessed a number of resistance genes including *bla*_CTX-M-15_. *bla*_NDM-5_ was located on a complex class 1 integron and adjacent to the IS*26*-mediated composite transposon carrying *mphA*-*mrx*-*mphR* in FUJ80154 (Fig. 1a). A similar structure has been found on an IncFIA/FIB plasmid (pNDM-5-1001) of an E. coli ST410 clinical isolate from China (12) and on an IncFII plasmid (pNDM-5-IT) of an E. coli ST167 clinical isolate from Italy (13), although there is a difference in the direction of the *mphA*-*mrx*-*mphR* module. Two copies of *bla*_OXA-48_, both located between two copies of *insA*-*insB* (IS*1R*), were present on the chromosome of FUJ80155 (Fig. 1b). The 2,666-bp composite transposon with IS*1R* at both sides of *bla*_OXA-48_ was also found in a Col156-type plasmid (pMTY17816_OXA48) harbored by a Klebsiella pneumoniae isolate (TUM17186) detected at a Japanese hospital from a patient with history of recent hospitalization in Vietnam (6). In both isolates (FUJ80155 and TUM17186), no direct repeat sequences were found adjacent to the region, which suggested that these isolates acquired the 2,666-bp genetic region by homologous recombination.

**FIG 2:**
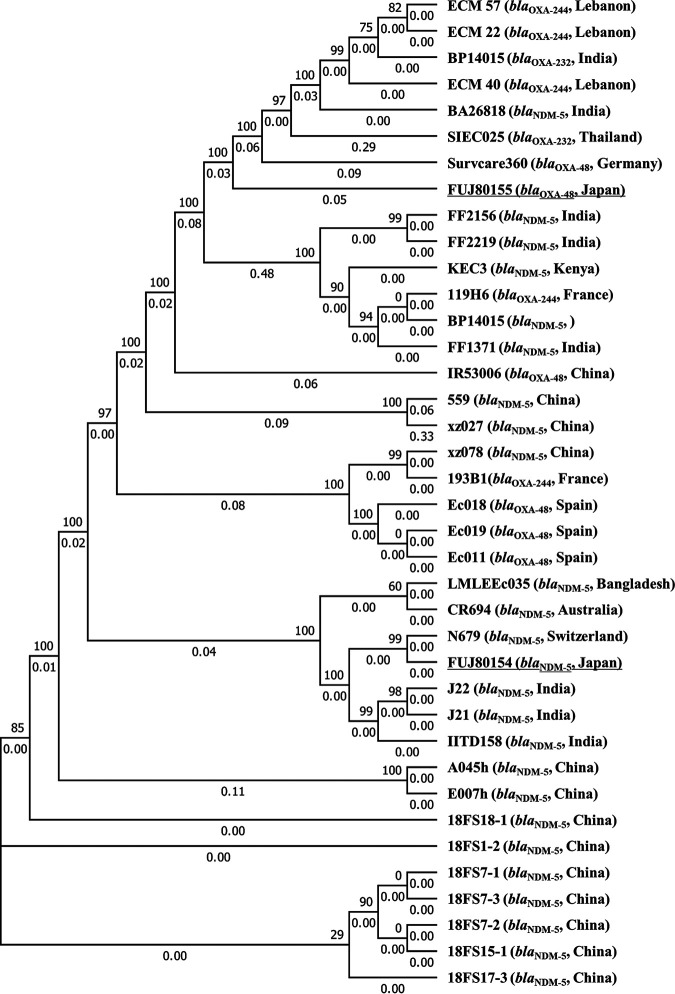
Phylogenetic tree of ST648 Escherichia coli isolates carrying *bla*_NDM-5_ or *bla*_OXA-48-like_. The carbapenemase genes carried by the isolates and the countries in which the isolates were detected are described in the parentheses after the isolate names. The isolates analyzed in this study are underlined.

While ST648 has been recognized as a major global clone of extended-spectrum β-lactamase (ESBL)-producing E. coli (14, 15), ST648 isolates carrying carbapenemase genes have also been reported. The first NDM-5-producing strain ever reported was ST648 E. coli, which was detected from a patient transferred directly from India to the United Kingdom (16). In another study, one of 10 NDM-1-producing E. coli isolates from the United Kingdom and two of seven NDM-1-producing E. coli isolates from Pakistan were ST648 (17). All of these isolates also carried *bla*_CTX-M_. OXA-48-producing E. coli ST648 isolates have been documented in several countries, all of which also produced CTX-M-15 (18, 19). One of two patients with OXA-48-producing E. coli ST648 in Poland had history of hospitalization in Cambodia, and the other had history of numerous foreign travels (18). These reports suggest that CTX-M-15-producing ST648, which has selective advantage on its own, has acquired carbapenemase genes that are spreading in each region, and is also beginning to spread across borders. As of 11 October 2021, whole genome data for 24 ST648 isolates carrying *bla*_NDM-5_ and 12 ST648 isolates carrying *bla*_OXA-48-like_ were available in GenBank (Table S2). The core-genome based-phylogenetic analysis of these genomes with those of the isolates analyzed in this study was performed using FUJ80155 as the reference genome as described previously (4). The phylogenetic tree was generated with FastTree 2.1.9 (https://bioweb.pasteur.fr/packages/pack@FastTree@2.1.9). Six isolates (LMLEEc035, CR694, N679, J22, J21, and IITD158) were closely related with FUJ80154 with 22–80 core genome SNPs (Fig. 2). Four of these isolates were from India and Bangladesh, and one of the remaining isolates from Australia was detected in a patient who had just returned from India (20). On the other hand, FUJ80155 had no closely related isolates with the SNPs to the closest isolate (ECM 22) being 2,147. This result supports the presumption that FUJ80155 independently acquired *bla*_OXA-48_ by homologous recombination.

In this study, CPE producing non-IMP carbapenemases, which are rare in Japan, was detected in two patients with a history of living abroad but no history of local hospitalization prior to the travel. Meanwhile, no CPE isolates were detected from routine screening of patients with history of inpatient care in foreign countries within 1 month of admission during the same period (data not shown). Although short-term travel has been reported to be a risk for acquisition of ESBL-producing *Enterobacterales*, acquisition of CPE was extremely rare in an analysis of the same travelers (21, 22). However, a recent French study demonstrated that international travel without history of hospitalization is a risk factor for detection of extensively drug-resistant organisms, such as CPE through inpatient screening (23). In recent years, community transmission of carbapenem-resistant Gram-negative organisms has been reported in several South and Southeast Asian countries, and it is assumed that the risk of acquiring CPE among travelers and residents without medical exposure is higher than before in these areas (24, 25). The reasons for the rarity of non-IPM carbapenemases in Japan are not clear, but the relative geographical isolation and low volume of cross-border traffic, along with early detection and isolation of patients carrying CPE of foreign origin may have contributed. As community transmission of CPE increases in some countries, international visitors without previous medical exposure who are currently not subjected to active screening may serve as a source of CPE that is uncommon locally.

In conclusion, E. coli ST648 isolates carrying carbapenemase genes were detected in two patients with a history of living abroad but with no history of local hospitalization. Although the cost of active screening and preemptive isolation must be balanced against the damage caused by the spread of MDROs, screening for MDROs on admission to hospital may be considered for international travelers and residents without history of hospitalization.

## Accession numbers.

Nucleotide sequences of the chromosome and plasmids of non-IMP CPE isolates have been deposited in the NCBI database under accession number AP024687-AP024693 (FUJ80154) and AP024694-AP024701 (FUJ80155).
